# Bloom filters for molecules

**DOI:** 10.1186/s13321-023-00765-1

**Published:** 2023-10-12

**Authors:** Jorge Medina, Andrew D. White

**Affiliations:** Department of Chemical Engineering, University of Rochester, Rochester, NY USA

**Keywords:** Bloom filter, Fingerprint, SMILES, Hashing

## Abstract

Ultra-large chemical libraries are reaching 10s to 100s of billions of molecules. A challenge for these libraries is to efficiently check if a proposed molecule is present. Here we propose and study Bloom filters for testing if a molecule is present in a set using either string or fingerprint representations. Bloom filters are small enough to hold billions of molecules in just a few GB of memory and check membership in sub milliseconds. We found string representations can have a false positive rate below 1% and require significantly less storage than using fingerprints. Canonical SMILES with Bloom filters with the simple FNV (Fowler-Noll-Voll) hashing function provide fast and accurate membership tests with small memory requirements. We provide a general implementation and specific filters for detecting if a molecule is purchasable, patented, or a natural product according to existing databases at https://github.com/whitead/molbloom.

## Introduction

With the growing scale of molecular screening, which now involves searching through billions of chemical structures, the processing times for querying extensive compound datasets have significantly increased [1, 2]. To address this, Bloom filters can compact any database just for membership verification.

The Bloom filter, a space-efficient and probabilistic data structure, was designed to ascertain whether an element belongs to a specific set. First proposed by Burton H. Bloom [3], this data structure has demonstrated exceptional value for large datasets, where traditional set membership testing methods would be excessively time-consuming. At its core, the Bloom filter utilizes a fixed-size (*m*) bit array to represent *n* elements, employing *k* hash functions to map each element to *k* positions within the array [3–5]. This allows Bloom filters to conduct set membership tests with low false positive rates while utilizing less time and space compared to traditional data retrieval techniques.

Originally applied in dictionaries and spell checkers [3, 6], Bloom filters allowed for the quick identification of words within a given vocabulary, where the only significant drawback was with fake positives when misspelled words were labeled as being correct. Over time, the scope of their applications broadened to encompass web searches such as Google Chrome’s former implementation of a Bloom filter to detect malicious URLs [7], among other use cases [8–10]. Concrete examples of the usage of bloom filters in chemistry workflows include exploration of the chemical space while asserting either commercial availability or neglecting patented chemicals, without needing memory intensive databases or external server dependencies. A real-life example can be found in ChemCrow[11], where bloom filters are used in the molecule recommendation setting, making sure recommended chemicals are purchasable without intensive memory requirement. As underscored by the Bloom Filter principle [5], ”Wherever a list or set is used, and space is at a premium, consider using a Bloom filter if the effect of false positives can be mitigated.”

Traditionally, molecules have been represented using structure-based fingerprints [12]. In this study, we built different bloom filters using the Coconut database [13] to compare the effectiveness of structure-based hashing with string hashes in the Bloom filter; we demonstrate that string hashing consistently outperforms its counterpart. To provide further context, Table 1 presents well-known chemistry databases, their approximate number of compounds, storage size required for text (SMILES) representation, and a comparison with a Bloom filter designed to store an equivalent number of molecules.

This study explores the use of Bloom filters in molecular databases. Although, we refer alternative data structures that offer functionalities that can either mitigate some limitations of Bloom filters or serve entirely different objectives. For example, Cuckoo filters [14] provide the capability for dynamic item insertion and deletion, a feature absent in conventional Bloom filters. Other alternatives, such as Quotient filters [15] and Count-Min sketches [16], also offer unique advantages and can be found in the literature. On a different note, Locality-Sensitive Hashing (LSH) [17] serves the specialized purpose of maximizing hash collisions to facilitate similarity searches. However, LSH techniques often grapple with computational challenges as data scales, leading to memory requirements that can quickly exceed available main memory. In contrast, Bloom filter indices, even for extensive databases like ZINC, can comfortably reside in the main memory of everyday household devices, such as smartwatches or cellphones.

**Table 1:** Examples of Chemical Compounds Databases

Name	Size (# of compounds)	Size (GB)$^a$	Bloom filter size needed (GB)$^{b,c,d}$
ZINC [2]	$>2$ billion	>100	>2.56730
ChemBL [18]	2, 354, 965	0.117	0.003023
Coconut [13]	407, 270	0.0204	0.000522
BindingDB [19]	566, 000	0.0283	0.000727
PubChem [20]	113, 993, 087	5.700	0.146344
SureChemBL [21]	22, 843, 364	1.1422	0.02932
Available Chemical Directory	$>3,2$ million	> 0.16	>0.004108
ChemNavigator	10, 000, 000	0.5	0.012837
ChemBridge	1,3 million	0.065	0.001668
ChemSpider [22]	$>115,000,000$	>5.75	>0.14763

$^a$ Estimated text file sizes for SMILES based on a 95 M sample; total sizes inferred by linear scaling to avoid loading entire datasets. $^b$ Estimated Bloom filter size needed for a fixed false positive rate of 0.005. $^c$ With this specifications, all Bloom filters use 8 hashing functions. $^d$ To get the length in bits of the filters transform from GB to bits with $1\texttt {GB} = 8 \times 10^{9} \texttt {Bits}$. Finally, the index time for our Bloom filters is approximately 1 million elements per second

A Bloom filter is initialized with an m-length bit vector, with all positions set to zero, and employs k independent hashing functions. These hashing functions generate k values ranging from 0 to m−1, which correspond to the positions in the bit vector where a ”1” will be assigned. The hashing functions must exhibit the following characteristics [23]: (1) Quick computation; (2) An avalanche effect, where minor input changes result in substantial and unpredictable output alterations, and (3) The generation of integers between 0 and m−1.

**Fig. 1:**
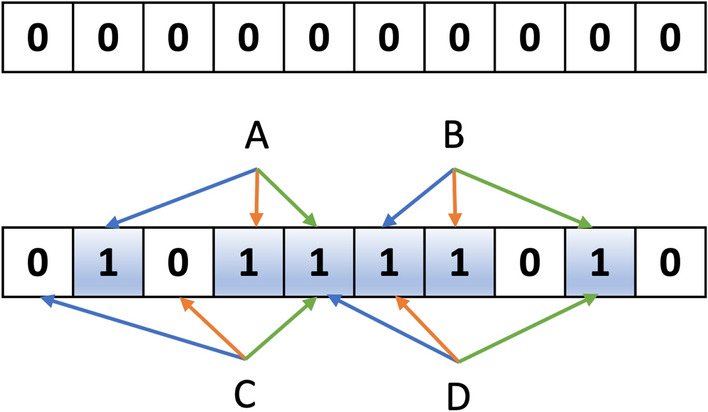
Scheme of Bloom Filters. In this generic Bloom filter example, we start with an empty bit array of zeros and four elements:** A**,** B**,** C**, and ** D**. The first two elements (A and B) are added to the filter, while the latter two (C and D) are queried. The process utilizes three distinct hashing functions, represented by colored arrows. To verify if elements C and D have been previously added to the filter, they are checked using these hashing functions. For element C, one of the hashing functions points to a zero bit, indicating that the element has not been added to the filter. However, all three hashing functions for element D point to bits already set to one, resulting in a false positive

**Fig. 2:**
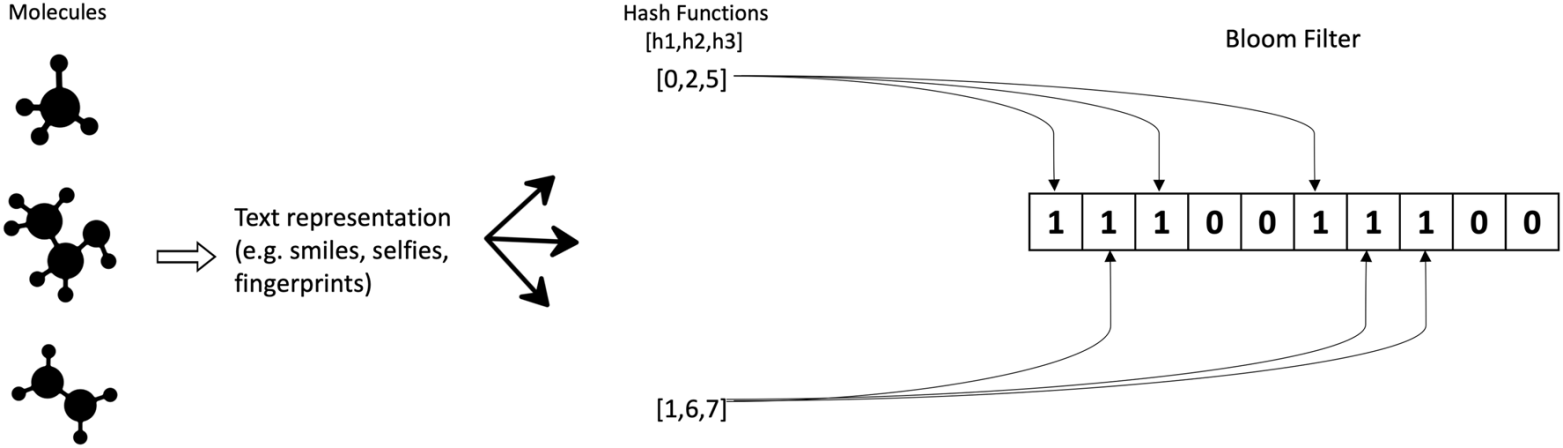
Illustrative Example for Bloom Filter with Molecules. When populating a Bloom filter, a set of molecules is initially stored in either a text format such as SMILES or SELFIES, or as fingerprints. Subsequently, distinct hashing functions generate indices (three in this example) for each element to fill the filter

Bloom filters enable the addition of new members but do not support individual removals. The filter can be queried to determine if a particular element has been added previously. However, this simplicity comes with certain drawbacks, such as the potential for two or more elements to be hashed to the same position in the Bloom filter (i.e., collisions). As a result, removing an element (by changing its positions from one to zero) could inadvertently affect other members with overlapping positions. This issue underscores the importance of randomness in hashing functions, often referred to as the avalanche effect. Figures 1 and 2 illustrate the workings of a Bloom filter and the storage of molecules within such filters.

For more comprehensive information on Bloom filter functionality, theoretical limits, and optimal implementation, refer to the existing literature. [3–5, 24]

Double hashing is employed to minimize the probability of collisions in the indexing of new members. Two distinct ”universal hashes,” $h_\alpha$ and $h_\beta$, are utilized to obtain k individual indices [25]:

$$h_i(A) = (h_\alpha (A) + i * h_\beta (A)) \ mod \ |m|$$

Here, ‘*A*’ represents an element being hashed, $h_i$ refers to one of the k hash functions generated per element (as illustrated in Figs. 1 and 2), |m| denotes the fixed size of the filter, and ”mod” signifies the remainder of the division. Restrictions that reduce collision probability are [25]:

- $$h_\beta \ne 0$$,
- $h_\beta (A)$ should not be divisible by the size of the filter.

Using the described method, the number of generated hashing functions can be selected depending on the number of elements to add (n), the bloom filter size in bits (M), and the pre-stablished false positive rate ($\epsilon$).

If the false positive rate is specified, M can be calculated as follows.

1 $${M = - \frac{n \ln \epsilon }{(\ln 2)^2} }$$

Conversely, altering M impacts the final false positive rate. Given M and the number of elements to be added, the number of hash functions k is calculated as:

2 $${k = \max \left( 8, \min \left( 64, \frac{M}{n * \log (2)}\right) \right) }$$

This will yield a range from 8 to 64 hashing functions.

## Methodology

The Python package MolBloom developed for this work [26] is an open-source package designed for molecules, featuring a built-in filter with ZINC-in-stock molecules. The package permits the creation of custom filters of varying sizes, which were adjusted in increments of one order of magnitude. Tests were conducted using the Coconut dataset [13] (approximately 400,000 molecules).

For comparative purposes, molecular fingerprints were employed to populate a Bloom filter and measure the false positive rate for increasing bit-array sizes. The hashing functions used in this study include Fowler-Noll-Voll (FNV) [27], as well as message digest 4 and 5 algorithms (MD4 and MD5) [28, 29] for string hashing. For chemical structure fingerprints, six combination between MACCS [30], Morgan [31], Atom-pair [32], and RDKit Fingerprints were utilized. This was done to investigate how traditional ways to hash molecules would act in this setting. FNV is a hash function designed for rapid, non-cryptographic hashing of data, leveraging prime numbers and bitwise operations to generate hash values that identify unique data elements. The FNV algorithm offers variants of different bit sizes and prime numbers, such as FNV-1 and FNV-1a. MD4 and MD5 are well-established hashing functions within the computer science community [33].

To assess false positive rates in each filter with different sizes, a fifty-fifty split was performed. The first half was added to empty filters, followed by membership testing in the second half. Any molecules from the second half classified as part of the set were counted as false positives.

An evaluation was conducted to compare the speed of Bloom filters and traditional methods in searching for elements within a dataset (using the dataset’s native API).

## Results and discussion

**Fig. 3:**
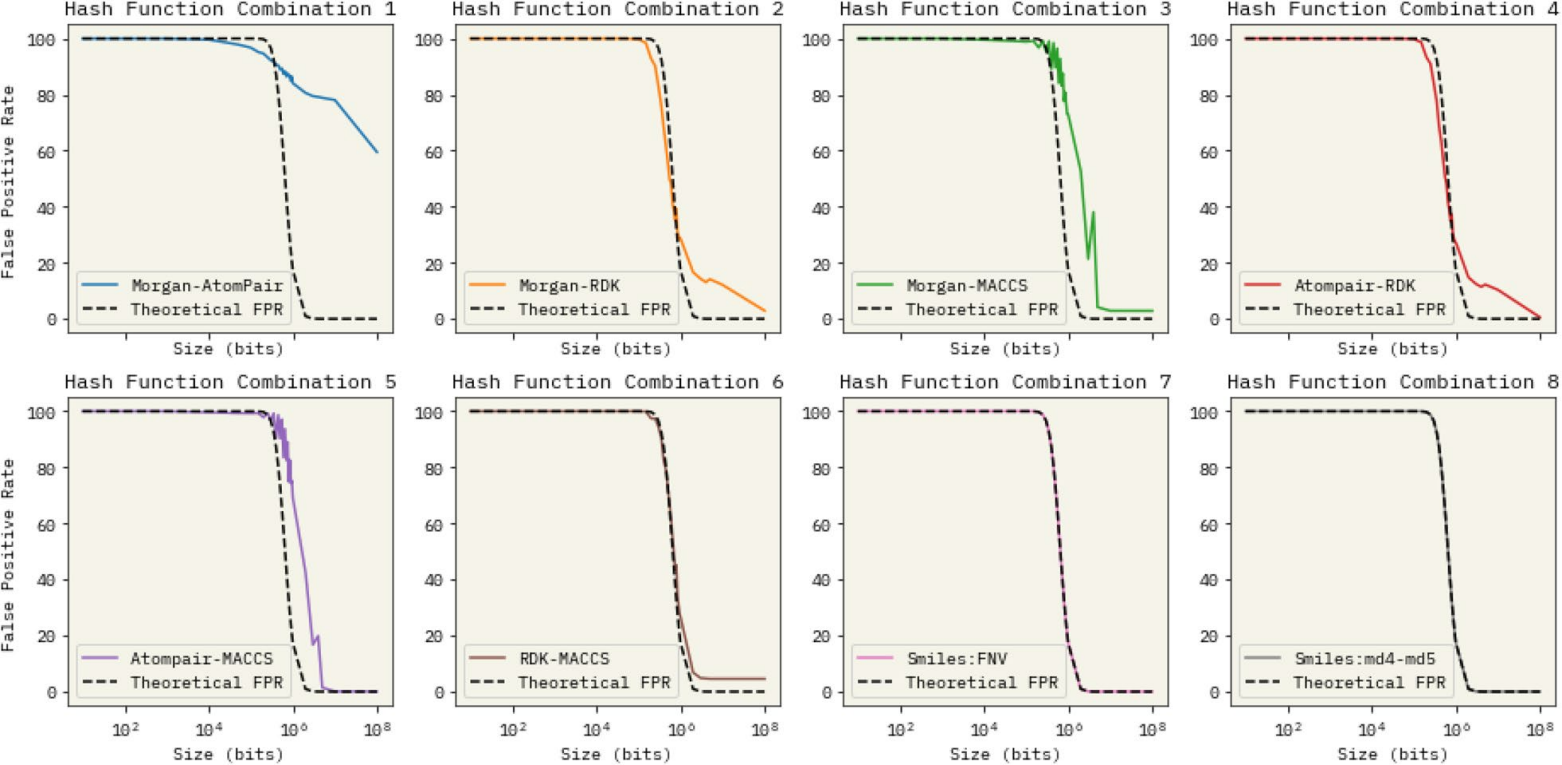
False Positive Rate (FPR) vs. filter size for different hash methods. Although all hashing variations follow similar trends, both string hashes FNV and MD4-5 are identical. “Noisy” peaks can be seen, which result from hashing functions being divisible by the size of the bloom filter

All six possible fingerprint combinations across eight distinct orders of magnitude for the Bloom filter and string hash implementations were examined. Figure 3 provide a comprehensive summary of the results.

As illustrated in Fig. 3, two key observations can be made. First, as anticipated, the false positive rate of Bloom filters approaches zero as the ratio between the filter size and dataset size increases. Second, the hashing of string SMILES representation outperforms most chemical structure fingerprints by over an order of magnitude in terms of false positive rate (combinations 7 & 8). Only the Morgan-MACCS and Atompair-MACCS fingerprint (combinations 3 & 5) hashing achieve false positive rates comparable to strings while requiring half an order of magnitude more bits of space.

Message Digest and FNV hashing (7 & 8) of strings yielded nearly identical and seemingly smooth curves, suggesting a well-randomized hashing of the elements. In contrast, other methods exhibit a “noisy” pattern, which serves as evidence of inadequate randomization. By design, these alternative methods are not highly randomized, as similar molecules tend to have comparable chemical fingerprints. This characteristic is the basis for their use in numerous optimization methods, as they can measure the distance between molecules. Consequently, their performance is suboptimal, as similar molecules have a higher likelihood of collisions within the Bloom filter.

**Fig. 4:**
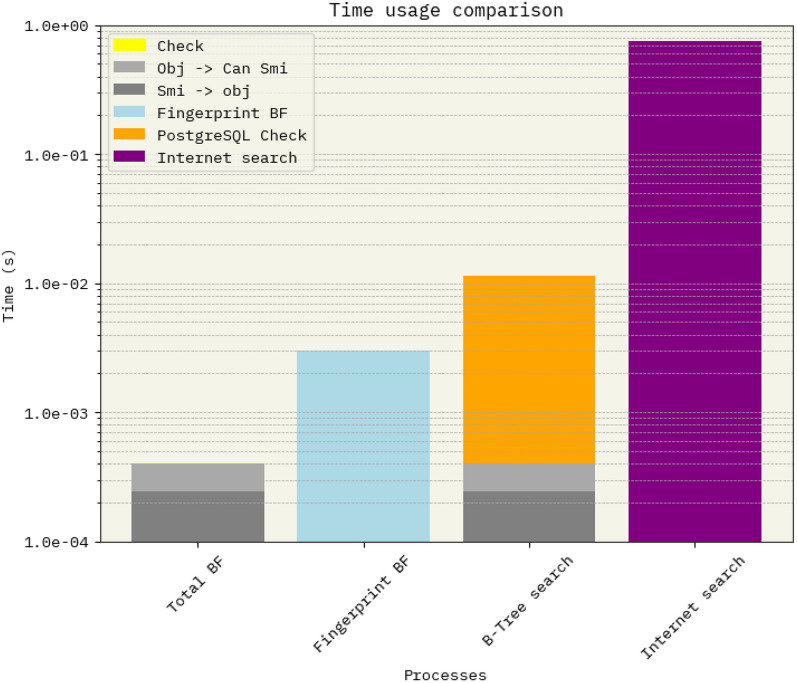
Comparison of Time Consumption Using Bloom Filters (BF), B-tree Indexing and Direct Server Search in a Commercial Database. The analysis for Bloom filters is broken down into individual steps:** a)** Conversion of SMILES notation to an RDKit Mol object,** b)** Canonicalization of the string. B-tree index was created with PostgreSQL. The time requirements for each step are displayed in the first and third columns. Bars 3 and 4, showcase how the Direct Server Search time is influenced by network latency, a common concern when relying on remote, online resources

In terms of the time required for Bloom filters to verify whether a molecule is part of a set or not, Fig. 4 clearly illustrates that Bloom filters demand up to three orders of magnitude less time compared to the native API, and one order of magnitude less than B-Tree indexing search. Even the “slower” Python implementation using RDKit for fingerprints necessitates two orders of magnitude less time for membership checks with an online server. To showcase the effect of latency in this test, a locally installed PostgreSQL database with a B-Tree index with 400,000 members was used. Assuming the Internet Search uses another efficient search method, the difference can be consider as latency.

## Conclusion

We demonstrate that string hashing (FNV and MD4–5) for Bloom filters outperform and approximate the theoretical limit of these structures, confirming that strings are sufficient for molecule storage. Even taking into account the time spent on canonicalizing SMILES, Bloom filter retrieval is still more than two orders of magnitudes faster than using an internet search. We also show that FNV, despite its simplicity and speed, is as effective as MD5. Employing other string representations, such as InChI and SELFIES, is expected to yield similar results. Potential applications for the Bloom filter are to quickly determine if a molecule is purchasable in ZINC[2], patented according to SureChembl [34], or a natural product [13].

## Data Availability

Molbloom package is an open source project and the code implementation in python for the experiments can be found in the corresponding repositories [26, 35]
